# Surgical management of renal cell carcinoma with subhepatic inferior vena cava tumor thrombus: a case report and review of the literature

**DOI:** 10.1186/s13256-024-04517-z

**Published:** 2024-04-23

**Authors:** Bekim Ademi, Luan Jaha, Isa Haxhiu, Xhevdet Çuni, Afrim Tahiri, Jetmir Gashi, Adhurim Koshi, Art Jaha

**Affiliations:** 1 Department of Vascular Surgery, University Clinical Center of Kosovo, Prishtina, Kosovo; 2 Department of Urology, University Clinical Center of Kosovo, Prishtina, Kosovo; 3 Department of Abdominal Surgery, University Clinical Center of Kosovo, Prishtina, Kosovo

**Keywords:** Renal cell carcinoma, Inferior vena cava involvement, Surgery

## Abstract

**Background:**

Renal cell carcinomas are the most common form of kidney cancer in adults. In addition to metastasizing in lungs, soft tissues, bones, and the liver, it also spreads locally. In 2–10% of patients, it causes a thrombus in the renal or inferior vena cava vein; in 1% of patients thrombus reaches the right atrium. Surgery is the only curative option, particularly for locally advanced disease. Despite the advancements in laparoscopic, robotic and endovascular techniques, for this group of patients, open surgery continues to be among the best options.

**Case report:**

Here we present a case of successful tumor thrombectomy from the infrahepatic inferior vena cava combined with renal vein amputation and nephrectomy. Our patient, a 58 year old Albanian woman presented to the doctors office with flank pain, weight loss, fever, high blood pressure, night sweats, and malaise. After a comprehensive assessment, which included urine analysis, complete blood count, electrolytes, renal and hepatic function tests, as well as ultrasonography and computed tomography, she was diagnosed with left kidney renal cell carcinoma involving the left renal vein and subhepatic inferior vena cava. After obtaining informed consent from the patient we scheduled her for surgery, which went well and without complications. She was discharged one week after to continue treatment with radiotherapy, chemotherapy, and immunotherapy.

**Conclusion:**

Open surgery is a safe and efficient way to treat renal cell carcinoma involving the renal vein and inferior vena cava. It is superior to other therapeutic modalities. When properly done it provides acceptable long time survival and good quality of life to patients.

## Background

Renal cell carcinomas (RCCs) in adults account for around 85% of kidney neoplasms [1, 2] and may be linked to various risk factors, such as genetics, smoking, obesity, and exposure to certain chemicals. Other potential risk factors include hypertension, exposure during work to trichloroethylene, benzene or herbicides, the use of nonsteroidal anti-inflammatory drugs, dialysis, hepatitis C infection, and kidney stones [3, 4].

RCC can remain clinically undetected throughout much of its progression. In around 90% of cases, RCC does not present with the hallmark symptoms of flank mass, hematuria, and flank pain until the disease has progressed significantly. Other signs and symptoms are weight loss, malaise, fever, night sweats, hypercalcemia, and hypertension. Male patients may also experience varicoceles on their left side as a result of obstruction of the testicular vein. However, almost one third of patients show no symptoms and end up discovering the carcinoma incidentally [5].

Around one third of RCC patients develop metastatic disease, with metastases being present either at the time of diagnosis or, in up to half of cases, later after a nephrectomy. The most common sites for metastatic disease are the lungs, soft tissues, bones, and liver, although the skin and central nervous system are also frequently affected [6].

A comprehensive diagnostic approach typically involves urinalysis, blood tests, renal and hepatic profiles, and imaging techniques, such as PET scans and angiotomography. Percutaneous core biopsy may also be performed to determine malignancy status.

RCC is staged using tumor, node, metastasis (TNM) classification and the American Joint Committee on Cancer (AJCC) staging system. Higher grade tumors are associated with poorer prognosis; inferior cava vein involvement is classified as stage III within these staging systems [6].

**Fig. 1:**
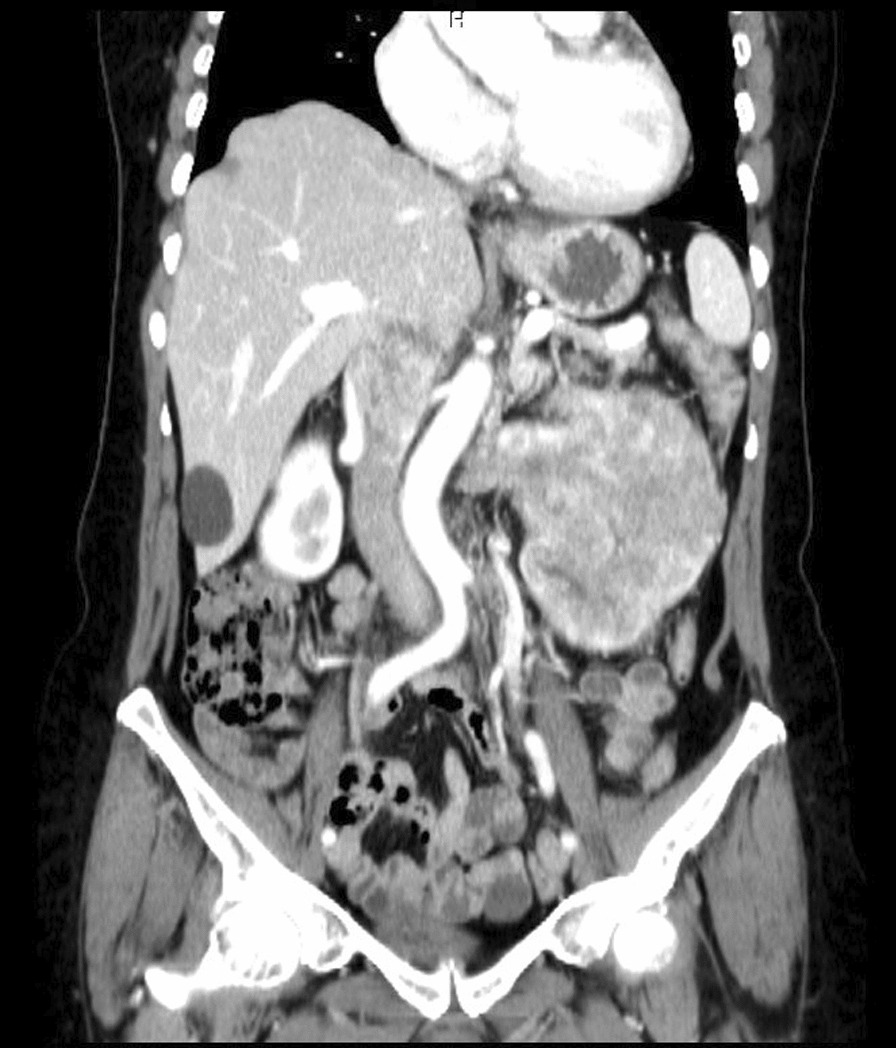
Preoperative computed tomography angiography, coronary view

Surgical intervention is currently the only effective treatment for localized RCC, although it may also be utilized to relieve symptoms in cases of metastatic disease. The specific surgical approach depends on the location of the tumor thrombus. Several surgical staging systems have been proposed, including the Neves, Novick, and Hinman systems. In the Novick system, which we have used in this case report, a tumor thrombus found in the renal vein that extends less than 2 cm within the inferior vena cava (IVC) is classified as a level I thrombus. An infrahepatic thrombus is classified as a level II thrombus. A level III classification is given to an intrahepatic IVC thrombus below the diaphragm, while a level IV classification is reserved for an IVC tumor thrombus that extends above the diaphragm, as illustrated in Fig. 1 [7–9].

## Case report

We are pleased to present a successful tumor thrombectomy from the infrahepatic inferior vena cava (class II) in patients with renal cell carcinoma. Our patient was a 58-year-old Albanian housewife who presented at the doctors office with a range of symptoms, including flank pain, hematuria, weight loss, fever, hypertension, night sweats, and malaise.

Despite these symptoms, she remained well-oriented to person, place, and time. Her vital signs were slightly altered, with a blood pressure of 150/95 mmHg, heart rate of 90 beats per minute, respiratory rate of 20 breaths per minute, and temperature of 38 °C. The skin appeared reddened, warm, and moist, without any lesions or rashes observed. The head was normocephalic and the neck was supple with no masses or lymphadenopathy. No visual deficits, ptosis, or facial asymmetry indicating cranial nerve pathology were noted. Muscle strength was 5/5 bilaterally, and sensations were intact to light touch and pinprick throughout. Reflexes were 2 and symmetric in all extremities, with no pathological reflexes present. Gait was steady and coordinated, without any observed abnormalities. Palpation over the left flank elicited tenderness, pain, and guarding, as did percussion. No abnormalities were heard on auscultation of the abdomen.

**Fig. 2:**
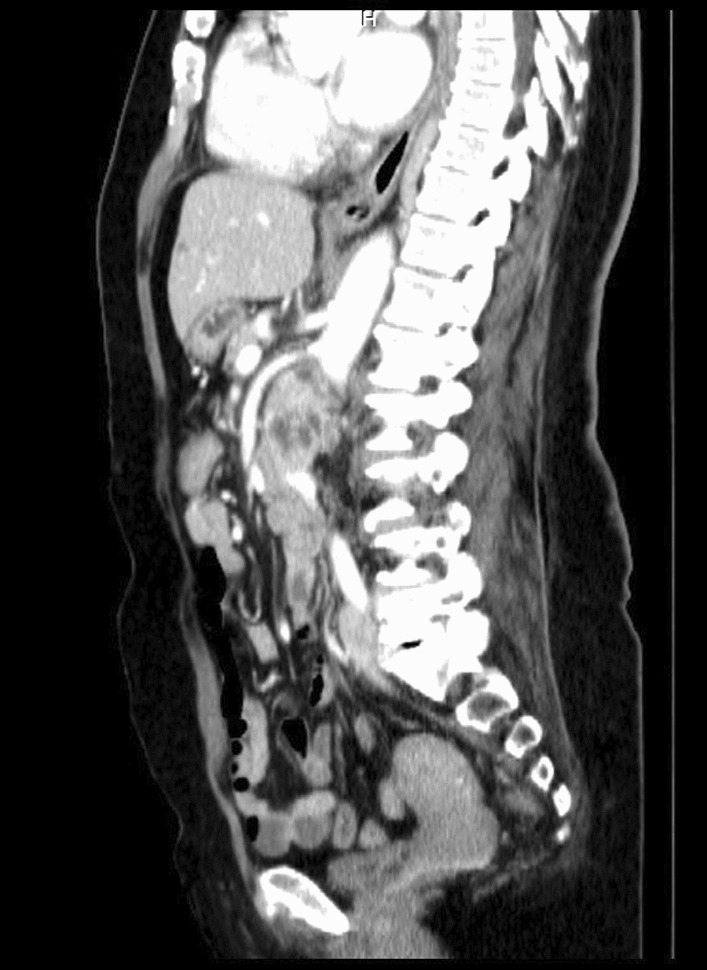
Preoperative computed tomography angiography, sagittal view

After a comprehensive assessment, which included urine analysis, complete blood count, electrolytes, renal and hepatic function tests, as well as ultrasonography and computed tomography, she was diagnosed with left kidney renal cell carcinoma involving the left renal vein and subhepatic inferior vena cava (Figs. 1, 2). All tests came back normal, except for microhematuria and slight hypoalbuminemia. A minor increase in C-reactive protein was also noted (Table 1).

**Table 1:** Blood test and urine analysis

Test	Value	Normal range
Blood analysis		
Erythrocyte sedimentation rate	15 mm/H	−15
Red blood cells	3.7 × 10^12^/L	3.8 – 4.9 × 10^12^/L
Hemoglobin	118 g/L	120–160 g/L
Hematocrit	37%	35–45%
White blood cells	9 × 10^9^/L	4 – 8 × 10^9^/L
Platelets	190 × 10^9^/L	140–310
Blood urea	5.2 mmol/L	2.5–8.3 mmol/L
Creatinine	80 mmol/L	44–88 mmol/L
Aspartate aminotransferase (AST)	< 37 µ/L	5–40 U/L
Alanine aminotransferase (ALT)	< 50 U/L	7–56 U/L
Total bilirubin	18 mmol/L	5.0–20.0 mmol/L
Direct bilirubin	< 7 mmol/L	< 7 mmol/L
Total protein	65 g/L	65–83 g/L
Albumin	36 g/L	35–53 g/L
C reactive protein	9 mg/L	0–6 mg/L
Cholesterol	3 mmol/L	< 6.5 mmol/L
Triglycerides	1.3 mmol/L	0.4–1.8 mmol/L
Urine analysis		
Color	Pale to dark	Yellow
Clarity	Cloudy	Clear or cloudy
pH	6	4.5–8
Specific gravity	1.010	1.005–1.025
Glucose	≤ 130 mg/dL	90 mg/dL
Ketones	None	None
Nitrites	Negative	Negative
Bilirubin	Negative	Negative
Urobilinogen	Negative	0.5–1 mg/dL
Blood	Positive	Negative
Urine sedimentation	40 RBC/hpf, 20–25 WBC/hpf, 15–20 squamous epithelial cells/hpf	

Apart from for a beta blocker (carvedilol 6.25 mg, twice a day orally) that she was taking for hypertension, she was on no other medications. The patient was a nonsmoker and did not consume alcohol. There was no history of kidney or other malignancy in the family.

The cancer extension into the left renal vein and subhepatic inferior vena cava corresponded to Neves II stage disease. After careful consideration, we decided to perform a nephrectomy, renal vein amputation and thrombectomy of the subhepatic vena cava.

A team composed of vascular surgeons, urologists, a hepatobiliary surgeon, and an anesthesiologist came together to form a multidisciplinary team. Adequate amounts of blood products, such as packed red blood cells, platelets, cryoprecipitate, fresh frozen plasma, and clotting factors, were made available for surgery. The surgical procedure was carried out under general endotracheal anesthesia. To monitor and resuscitate the patient during surgery, a large bore central venous catheter, a 15 F catheter in the right internal jugular vein, and a right radial arterial catheter were inserted.

**Fig. 3:**
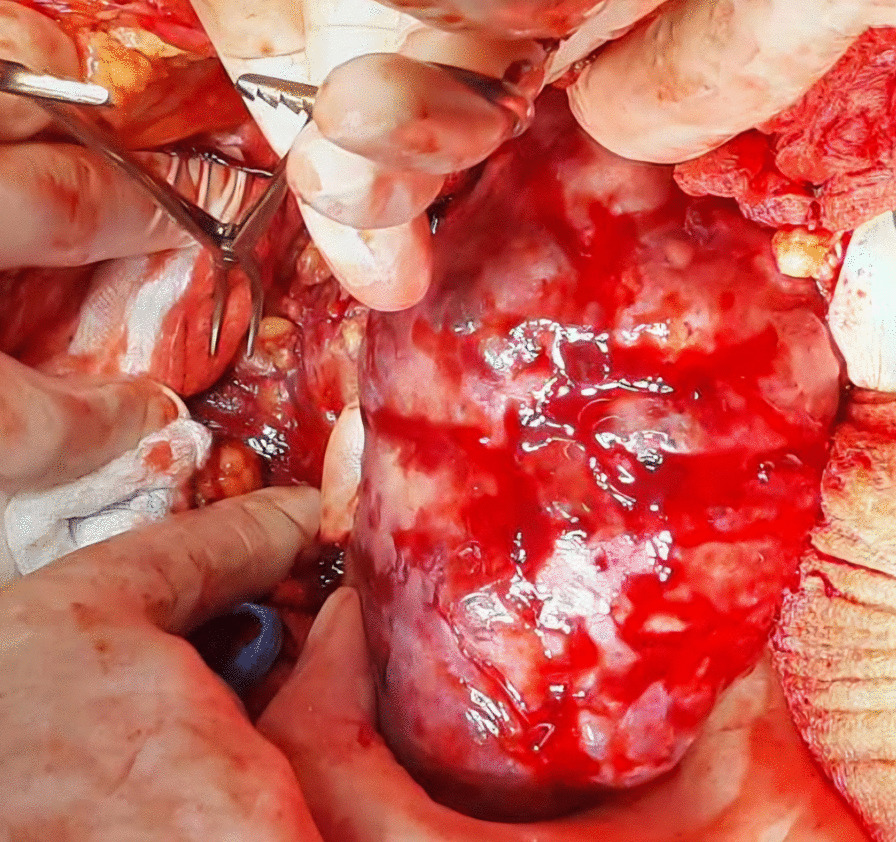
Nephrectomy

A midline incision was made to approach the abdomen as it was thought to provide optimal exposure of the inferior vena cava and contralateral kidney, enable thorough metastatic evaluation, and minimize postoperative pain. Since we decided to remove the kidney first and cancer thrombus after, we mobilized the colon medially, brought the kidney outside of Gerota’s fascia, and tied the ureter. After tying the renal artery and leaving the kidney attached only by the renal vein, the kidney was removed (Fig. 3).

**Fig. 4:**
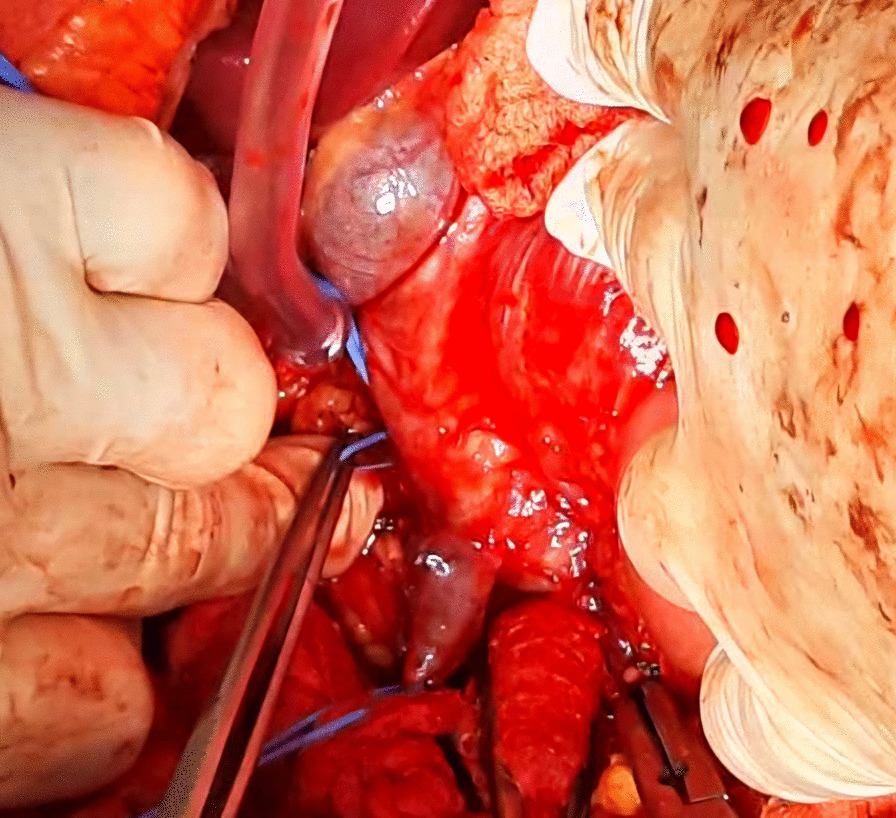
Vascular control of the inferior vena cava, right renal vein, and ovarian vein

**Fig. 5:**
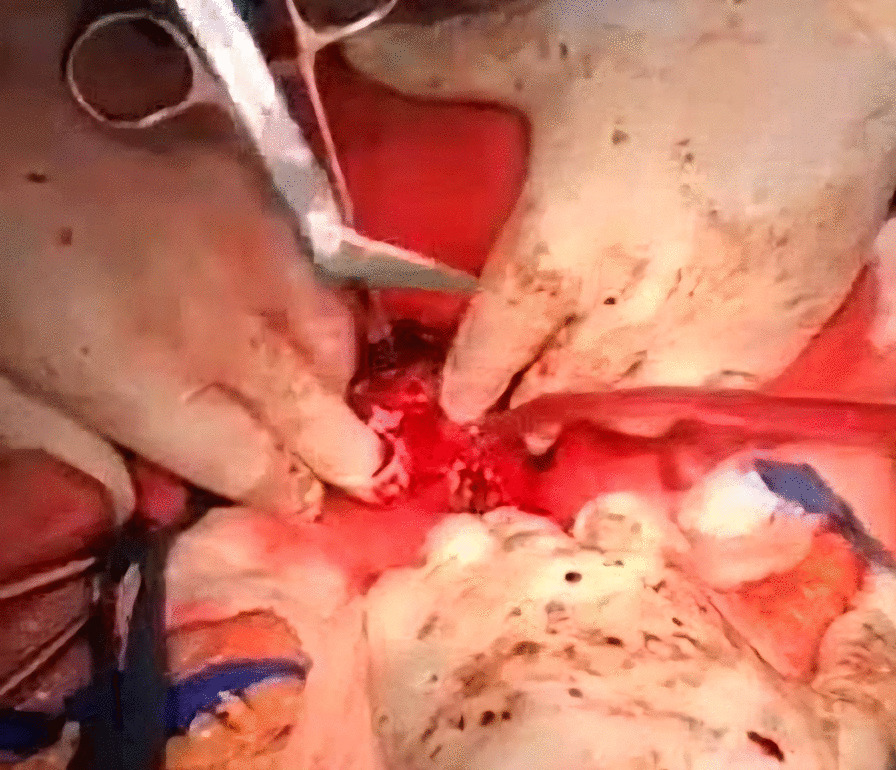
Cavotomy

**Fig. 6:**
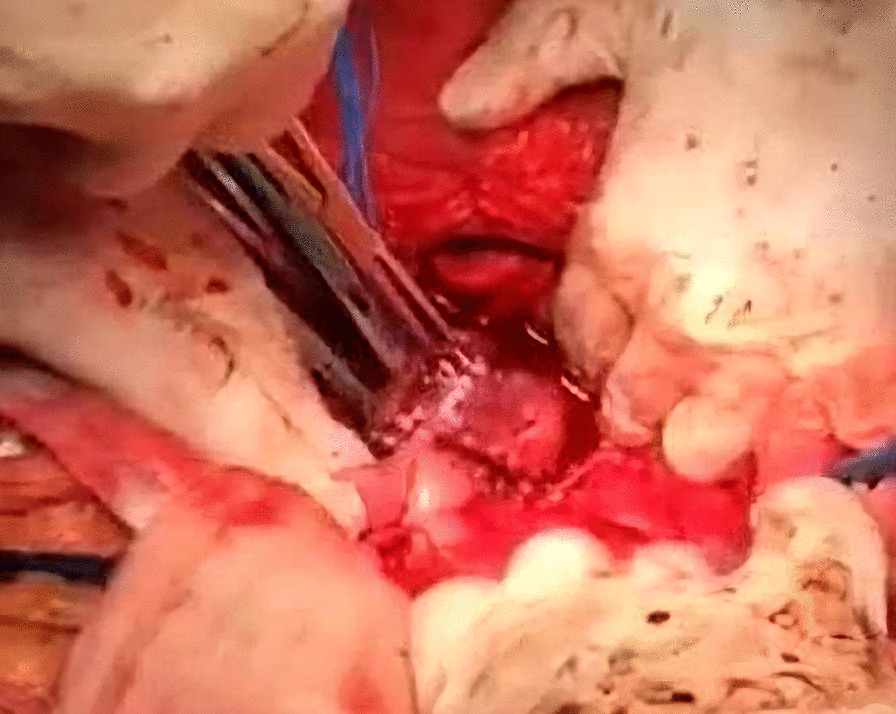
Tumor thrombus removal

**Fig. 7:**
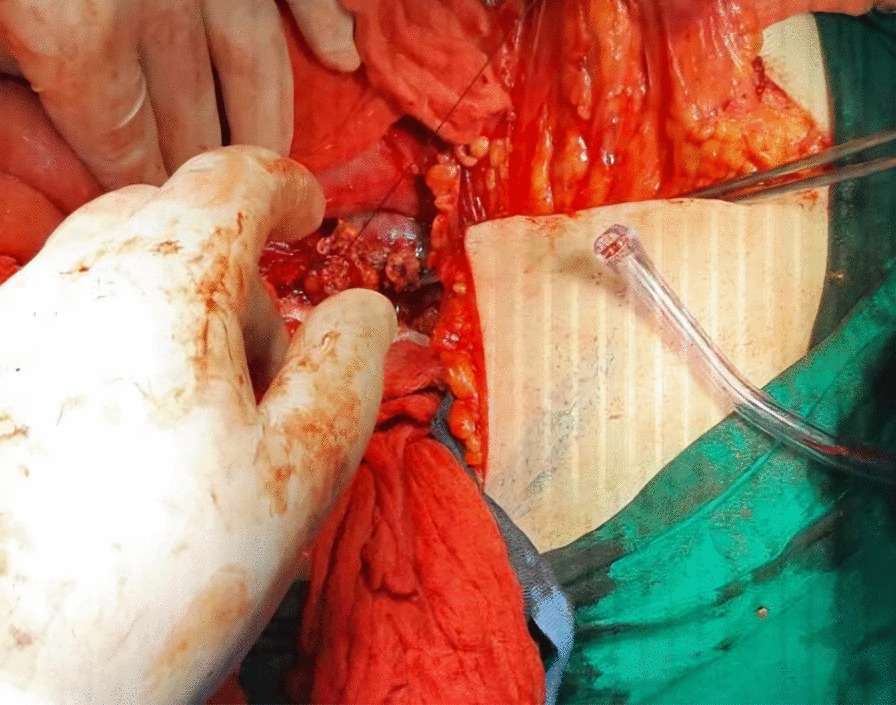
Suturing of the inferior vena cava

Afterwards, liver was separated from its connections and tourniquets were positioned around the suprahepatic IVC, which was then mobilized together with the contralateral renal vein. To manage and identify lumbar veins, vascular clamps were placed above and below the tumor thrombus (Fig. 4). Patient response was monitored with a 1 minute test clamp, during which no hemodynamic changes were observed, allowing the clamps to remain in place as we performed cavotomy. A precise incision was created around the ostium of the renal vein into the IVC, followed by placement of a Fogarty catheter to facilitate thrombus removal (Fig. 5). The tumor itself was successfully removed without any issues and the cavotomy incision was closed using a 3–0 polypropylene suture (Figs. 6, 7). After ensuring meticulous hemostasis, the abdominal cavity was closed using multiple layers.

**Fig. 8:**
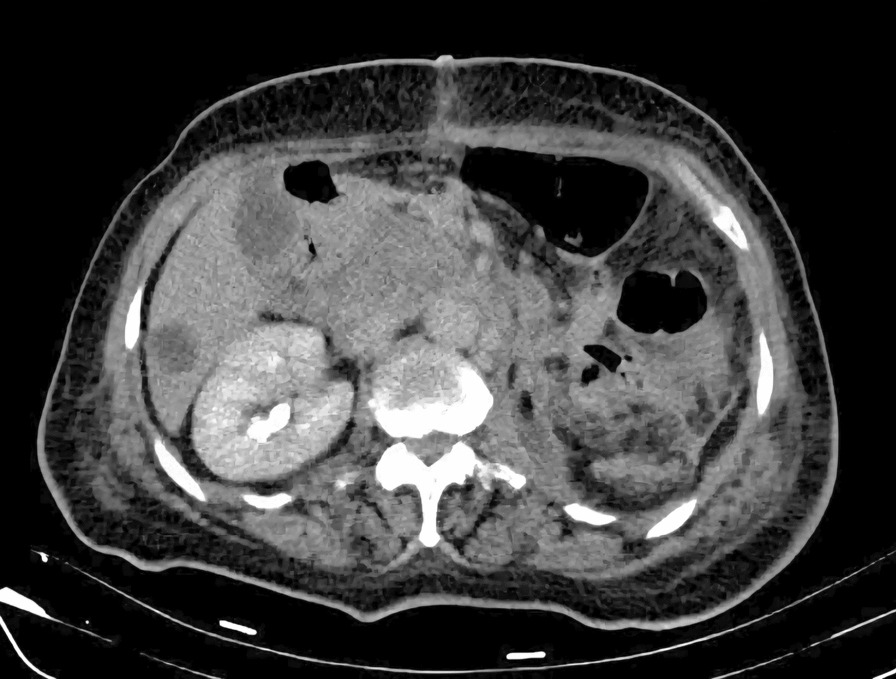
Postoperative computed tomography

Overall, the patient received 10 units of packed red blood cells, 10 units of fresh frozen plasma, 10 units of platelets, and 10 units of cryoprecipitate. The surgery proceeded without complications. The patient was then referred for further treatment in the ICU, where she stayed for a day for postoperative care. She received an additional two units of red blood cells and one unit of plasma to achieve a hematocrit value of 38% and hemoglobin level of 115 g/L. Blood urea nitrogen (BUN) and creatinine levels were within the normal range, as were the electrolyte levels. Liver enzyme levels were also normal. Hemodynamically, she remained stable, and her oxygen saturation consistently stayed above 98%. At 6 hours later, she was successfully extubated. On the second day after surgery, she was transferred to the ward, and 1 week later, she was discharged to continue treatment with radiotherapy, chemotherapy, and immunotherapy. During her ward stay, she received intravenous antibiotics (1 g of ceftriaxone twice daily and metronidazole 500 mg three times daily), subcutaneous low molecular weight heparin (enoxaparin 4000 IU/day), intravenous proton pump inhibitor (pantoprazole 40 mg once daily), and analgesics (diclofenac 50 mg intravenously twice daily for the first two days and as needed for pain thereafter). No additional blood transfusions were required (Fig. 8).

The ICV is free of thrombus, but there are already developed regional and liver metastases.

On the follow-up computed tomography (CT) scan performed one month after surgery, the inferior vena cava was free of tumor, but unfortunately, the tumor had already spread to the liver and retroperitoneum (Fig. 7). The patient’s condition deteriorated 3 months after surgery and despite aggressive chemotherapy, she passed away 6 months later. Owing to religious reasons, no autopsy was performed.

## Discussion

In the midst of the ongoing debate concerning the role of open surgery in the treatment of renal cell carcinoma involving the renal vein and inferior vena cava, especially when compared with less invasive surgical approaches, our case presentation aims to align with those who consider open surgical thrombectomy from the infrahepatic inferior vena cava combined with renal vein amputation and the removal of the affected kidney, a viable treatment option.

However, it requires extensive preoperative management, a multidisciplinary team, precise technical expertise during the procedure, exceptional anesthesia management, and diligent postoperative care. The surgical team must be well-prepared for any unexpected situations that may arise during the procedure while also having a clear understanding of the procedure at hand. When dealing with cases above the liver, the team should include vascular, urology, hepatobiliary, and cardiac surgeons. Additionally, strategies, such as kidney immobilization and tyrosine inhibitors, are recommended to decrease the size of the tumor before surgery.

The first routine preoperative arterial embolization of the kidney to decrease the tumor size and facilitated the surgical procedure was advocated by Pouliot and coworkers [10], but was found to have no significant advantage over no embolization. Preoperative arterial embolization of the kidney has not shown significant advantages and is only recommended in specific situations, as it is associated with complications, such as angioinfarction syndrome and inflammation around the kidney and surgical field [11]. The usefulness of tyrosine kinase inhibitors is still debated and remains to be confirmed through randomized trials despite its effectiveness in treating metastatic RCC [12–14].

There have been reports of surgeons placing IVC Greenfield Filters during surgery to prevent pulmonary embolism, but studies have found this practice to be ineffective and potentially dangerous. The filter may become clogged with thrombus, requiring complete removal and IVC reconstruction [15].

When dealing with a patient with RCC and IVC tumor thrombus, the surgical approach must be personalized on the basis of the level of the thrombus and characteristics of the tumor. Precise preoperative imaging plays a critical role in effective planning [16]. It is imperative to ascertain the location of the tumor thrombus—whether it is infrahepatic, intrahepatic, or suprahepatic—as this will dictate the appropriate surgical approach, methodology for controlling the inferior vena cava, and potential requirement for vascular bypass [6].

In patients with RCC and IVC tumor thrombus, general endotracheal anesthesia is favored over regional epidural anesthesia owing to the risk of significant blood loss and coagulopathy, which increases the potential for epidural hematomas.

Various modes of surgical treatment have been described for patients with RCC involving renal vein and IVC, including laparoscopic, robotic, and endovascular surgery [17–20]. In certain exceptional situations of renal cell carcinoma (RCC) and tumor thrombus in level I of the inferior vena cava (IVC), a laparoscopic method might be feasible. However, recorded cases have indicated an elevated possibility of intraoperative complications with minimal proof regarding its cancer-fighting efficacy and safety. Moreover, robotic radical nephrectomy has proven to be effective in certain instances of low-level thrombi, but thorough research remains inadequate, and these minimally invasive procedures are not commonly recommended [6].

Open surgery, specifically nephrectomy and IVC thrombectomy, remains the preferred option and has shown usefulness even in metastatic disease, provided the patient is a candidate for surgery and has local symptoms [21]. In patients with metastatic RCC, the most reliable indicators of their prognosis are their response to systemic therapy and the burden of their metastatic disease [22–24].

The factors that could affect the survival of patients in this cohort are related to pathological TNM stage, nuclear grade, histological tumor subtype, regional lymph node status, and perinephritic fat invasion. It is worth noting, however, that there is little or no correlation between the level of IVC tumor thrombus and overall survival (OS). According to a recent study, the recurrence rate might be influenced by the extent of IVC tumor thrombus, however, there does not seem to be any correlation with OS [25]. Additionally, research on the results of surgery in patients with RCC who underwent nephrectomy and IVC thrombectomy revealed that the existence of fragile venous thrombus could augment the probability of synchronous nodal or distant metastases [41]. According to reports, the 5-year OS rate following surgical approach ranges from 32 to 69% in patients with IVC tumor thrombus wall invasion [25–28].

As for the procedure itself, radical nephrectomy with concomitant IVC thrombectomy has a reported perioperative mortality rate of 5–10%. However, it has been associated with significant morbidity, resulting in an overall complication rate of 38% [50]. As such complex surgeries require extensive vascular, hepatobiliary, cardiothoracic, and anesthesia support, it is recommended that only centers of excellence with the necessary resources undertake these procedures. Specifically, they should be reserved for patients with more advanced level III and IV IVC tumor thrombi [29–32].

## Conclusion

The effective management of renal cell carcinoma and inferior vena cava tumor thrombus relies heavily on a skilled multidisciplinary surgical team. Adequate preoperative imaging is crucial for the planning and facilitation of surgical procedures for such cases. The surgical strategy should be customized to suit the specific characteristics and extent of the tumor thrombus situated in the inferior vena cava. While minimally invasive surgical techniques (including robotics) may have a role in this matter, their use should be limited to specific cases, particularly level I IVC tumor thrombus cases with favorable anatomic and tumor characteristics. For smaller lesions in patients who are not candidates for surgery, thermal ablation may provide a viable alternative. Metastatic disease in RCC can be treated with targeted therapy and immunotherapy, whereas chemotherapy and hormonal therapy have generally been unsuccessful.

## Data Availability

The data are available under consideration of the corresponding author upon reasonable request.

## Abbreviations

RCC: Renal cell carcinoma
IVC: Inferior vena cava
AJCC: American Joint Committee on Cancer
